# In Vitro Feasibility Analysis of a New Sutureless Wound-Closure System Based on a Temperature-Regulated Laser and a Transparent Collagen Membrane for Laser Tissue Soldering (LTS)

**DOI:** 10.3390/ijms21197104

**Published:** 2020-09-26

**Authors:** Moritz Alexander Birkelbach, Ralf Smeets, Imke Fiedler, Lan Kluwe, Martin Wehner, Tilmann Trebst, Philip Hartjen

**Affiliations:** 1 Department of Oral and Maxillofacial Surgery, University Medical Center Hamburg-Eppendorf, 20246 Hamburg, Germany; r.smeets@uke.de (R.S.); kluwe@uke.de (L.K.); p.hartjen@uke.de (P.H.); 2 Department of Oral and Maxillofacial Surgery, Division of Regenerative Orofacial Medicine, University Hospital Hamburg-Eppendorf, 20246 Hamburg, Germany; 3 Department of Osteology and Biomechanics, University Medical Center Hamburg-Eppendorf, 20246 Hamburg, Germany; i.fiedler@uke.de; 4 ILT, Fraunhofer-Institute for Laser Technology, 52074 Aachen, Germany; martin.wehner@ilt.fraunhofer.de; 5 LifePhotonic GmbH, 53129 Bonn, Germany; trebst@lipho.eu

**Keywords:** biocompatibility, in vitro, collagen membrane, sutureless wound closure, intraoral laser tissue soldering (LTS)

## Abstract

For the post-surgical treatment of oral wounds and mucosal defects beyond a certain size, the gold standard is still an autologous skin or mucosal graft in combination with complex suturing techniques. A variety of techniques and biomaterials has been developed for sutureless wound closure including different tissue glues or collagen patches. However, no wound covering that enables for sutureless fixation has yet been introduced. Thus, a new system was developed that allows for sutureless wound covering including a transparent collagen membrane, which can be attached to the mucosa using a specially modified 2λ laser beam with integrated temperature sensors and serum albumin as bio-adhesive. The sutureless wound closure system was tested for its applicability and its cytocompatibility by an established in vitro model in the present study. The feasibility of the laser system was tested ex vivo on a porcine palate. The in vitro cytocompatibility tests excluded the potential release of toxic substances from the laser-irradiated collagen membrane and the bio-adhesive. The results of the ex vivo feasibility study using a porcine palate revealed satisfactory mean tensile strength of 1.2–1.5 N for the bonding of the membrane to the tissue fixed with laser of 980 nm. The results suggest that our newly developed laser-assisted wound closure system is a feasible approach and could be a first step on the way towards a laser based sutureless clinical application in tissue repair and oral surgery.

## 1. Introduction

Larger mucosal defects, which can occur due to a variety of pathologies such as resections of different tumors or infections require for specialized techniques for wound closure. The gold standard treatments are distant or local flaps such as split and full-thickness skin grafts, oral mucosa free grafts, or oral connective tissue grafts [1,2,3]. However, these treatment options require initiation of a second surgical site and involve the associated risk in wound closure [3]. Moreover, varying success rates have been reported for these techniques [4].

Wound patches based on xenogeneic collagen have already been identified as a reliable treatment option [5,6]. In this context, it has been shown that collagen as a biomaterial supports the wound healing process due to its non-inflammatory properties and excellent biocompatibility and additionally collagen materials can promote epithelial regeneration [5,7,8,9]. Furthermore, the application of collagen materials has been shown to minimize scarring as it provides a structure for ingrowth of local cells and can constitute a sufficient barrier and protection against bacterial invasion [1]. Additionally to the graft, special suturing techniques must be applied to fixate the flaps or wound dressing materials in the majority of cases of such larger mucosal defects [10]. However, the complexity of these suturing techniques can lead to delayed or distorted mucosal healing due to an increased risk of treatment errors [11].

Besides, suturing into the oral cavity is partially hampered by the morphology of the tissue, e.g., suturing to the palatal late anchoring points. Alternatively, laser-mediated joining of tissues, which is termed ‘laser tissue soldering’ (LTS), has been proposed as a substitute for suturing or staples [12,13]. Interestingly, LTS is already used in different clinical indications because it additionally allows for the immediate sealing of wounds while immediately withstanding a much higher leakage pressure than suturing [14]. For example, LTS is used for the sealing of eye’s incisions which is important to prevent leakage due to the intraocular pressure [15,16,17]. Another application for LTS is the wound closure of skin or mucosa incisions, which has already successfully been investigated in vitro and in vivo [18,19,20,21].

For such indications, it has been demonstrated that the tensile strength measured directly after laser soldering can be as high as the strength obtained by suturing [22]. Even fixation of wound dressings on extended defects as burn wounds becomes feasible without the cosmetic drawbacks associated with staple marks and suturing [23]. Altogether, this procedure is expected to introduce less foreign body reactions than suture materials and improve wound healing [15,22]. In this context, it is known that the laser-generated fixation is based on a thermally-induced denaturation of a protein solution with correlated changes of the secondary and tertiary structure of proteins within the tissue. Thus, it is possible to utilize blood serum proteins such as albumin as bio-adhesives [24,25]. Typically, LTS includes the irradiation and heating up only for a few seconds of duration to limit the depth of the heat-affected zone and related tissue defects [26,27,28]. The irradiation depth depends strongly on the optical properties of the respective tissue which can also vary in different zones [29,30]. Thus, it is also not possible to use a standardized laser with one static protocol for one tissue as it has to be adaptable to the local tissue characteristics. For overcoming this issue, the use of temperature sensors into the laser device was proposed to measure the temperature during the LTS treatment and to control laser power accordingly to the local tissue characteristics [28].

The aim of the present study was the development of a new sutureless wound-closure system based on a temperature-regulated laser and a collagen membrane for oral wound closure. In this new system, the laser should be used as fixation device for a newly developed transparent collagen membrane. For this purpose, a laser device equipped with a handpiece, integrated temperature sensors and two diodes with wavelengths of 980 nm and 1470 nm was constructed. Moreover, a collagen membrane based on porcine pericardium was chosen [31], that most importantly was transparent since it was crucial that it was permeable or the laser beam. Additionally, it needed to be characterized with adequate properties regarding stability, biocompatibility, and degradability. Finally, collagen is an already well established biomaterial for oral wound treatment [32] and natural collagen is an important factor in all stages of wound healing that serves as a key extracellular component for repair and remodeling [33].

In the present study, the feasibility and the cytocompatibility of the components of this novel fixation method were assessed. The initial feasibility study included the fixation of the collagen membrane to the surface of a porcine palate using bovine serum as the bio-adhesive and measurements of the tensile strength to test the fixation-stability between the membrane and the tissue. In a second step, the cytocompatibility of the components was determined using ISO 10993-5/-12 based methods to exclude potential release of toxic substance from the biomaterials upon laser-irradiation [34].

## 2. Results

### 2.1. Structural Analysis of the Membrane 

**Figure 1:**
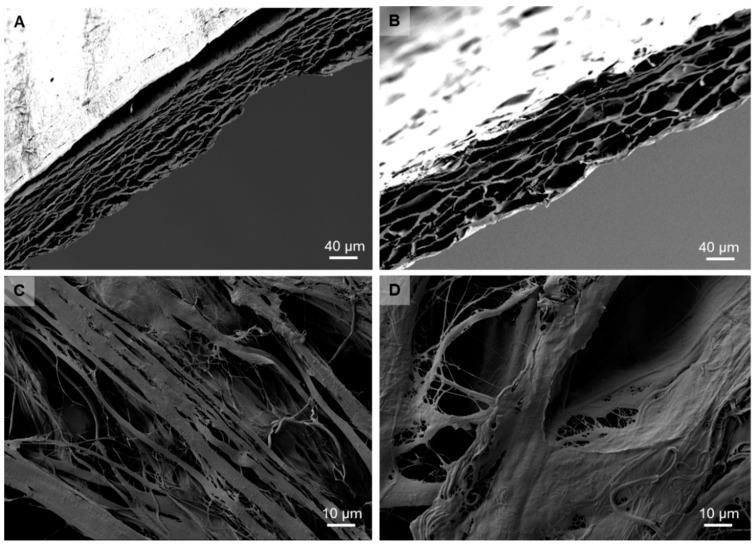
SEM images of the newly developed transparent collagen membrane (**A**,**C**) and the non-transparent collagen membrane (**B**,**D**). (**A**,**B**) show cross sections of both materials that revealed the collagenous nature of both biomaterials (500× magnification). (**C**,**D**) show the fibrillar surfaces of both materials (2000× magnification).

The analysis of the membrane properties showed that the newly developed transparent collagen membrane provided a material structure comparable to a native barrier membrane also based on porcine pericardium (Figure 1). Both membranes showed a layered structure, which is comparable to the collagen fiber bundles of the origin tissue (Figure 1A,B). Both surfaces showed a fibrillar microstructure (Figure 1C,D). No cells or remaining cell remnants were observable (Figure 1).

### 2.2. Ex Vivo Feasibility Test

The laser device allows several settings such as maximum surface temperature of the treated spot, maximum initial laser power for each wavelength and overall gluing time. It includes a temperature control system that was described by Wehner et al. [30]. It also allows a delayed start of the emission for one of the laser sources. Prior to the experiments described here, we gathered optimum parameters for the laser device and the gluing process of collagen membranes on various tissue samples from porcine cheeks and palate as described in the materials and methods section. Upon irradiation, the adhesive between the dressing and the tissue welded and the collagen membrane with dimensions of 10 × 15 × 0.05 mm was fixed to the underlying tissue at six points. The averaged diameter of the bio-adhesive spots was 4 mm. An overall gluing time of 6 s was sufficient, as well as starting the gluing process with 27 W of 980 nm and 6 W of 1470 nm laser power. We obtained higher tensile strengths when increasing the maximum surface temperature of the treated spot to 90 °C.

**Figure 2:**
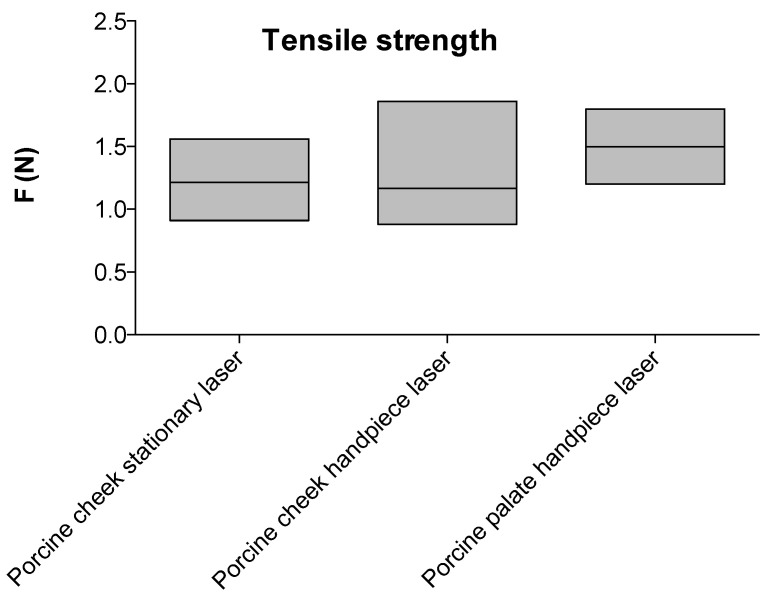
Tensile strength of the collagen membrane tested on porcine cheek and a porcine palate using a stationary laser and a laser that was equipped with a handpiece. The central lines represent means, the boxes represent minimum and maximum values.

The general feasibility of the welding process (fixation of the membrane on porcine intraoral tissue) was first tested in a stationary experiment on porcine cheek. The mean perpendicular tensile strength in this setup was 1.2 N (range 0.9–1.6 N) (Figure 2). A similar tensile strength was achieved using a laser that was equipped with a handpiece that was used freehandedly by the surgeon (1.2 N, range 0–9–1.9 N). A slightly higher tensile strength was achieved for porcine palate using the laser handpiece (1.5 N, range 1.2–1.8 N).

### 2.3. In Vitro Cytocompatibility Assessment

**Figure 3:**
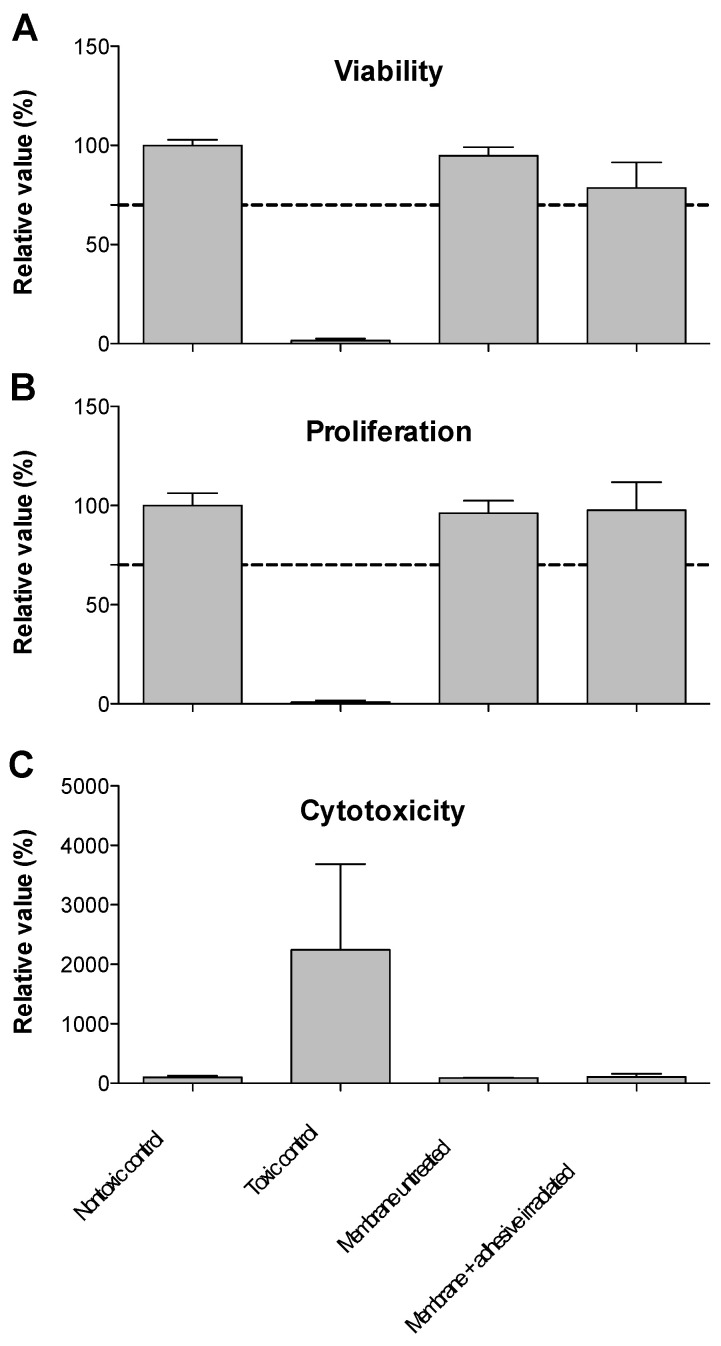
Cytocompatibility of the collagen membrane and the laser-welded membrane plus bioadhesive. (**A**) Viability, (**B**) proliferation, and (**C**) cytotoxicity. Values were normalized to the mean value of the non-toxic control. Columns represent means and the error bars depict the standard deviation. Dotted lines in (**A**,**B**) show 70% of the negative control which indicates the nontoxic range as defined in DIN EN ISO 10993-5:2009 [35].

Viability and proliferation (Figure 3A,B) of cells cultured in media incubated for 3 days with the membrane/bio-adhesive with and without laser irradiation were comparable with those of cells grown in media without contact to the membrane (Figure 3C). This indicates a lack of release of toxic components from the collagen membrane and the bio-adhesive, regardless of laser-irradiation. In concordance, no cytotoxicity was measured for any of the specimens (Figure 3C).

**Figure 4:**
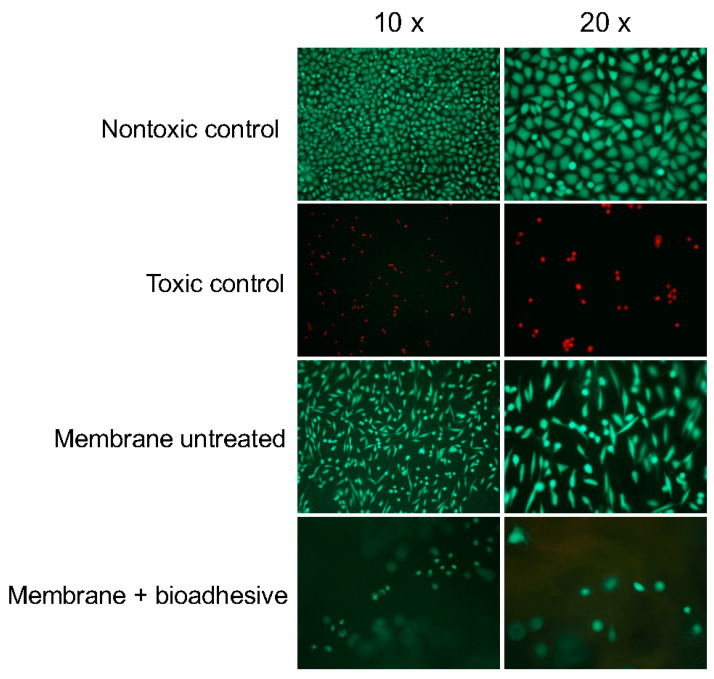
L929 cells grown on the collagen membrane and on the laser-welded membrane + bioadhesive. Vital and dead cells show green and red fluorescence, respectively. Fewer cells on the laser-welded membrane and lack of focus were due to laser-induced unevenness of surface.

When seeded directly onto the collagen membrane, large numbers of vital (green) and no dead (red) cells were visible (Figure 4). In addition, the cells exhibited a spindle-shaped morphology, similar to that of cells on the non-toxic control surface, indicating vitality and firm attachment of the cells. On the laser-irradiated collagen membrane with the bio-adhesive, cells appear rounder and were mostly out of focus due to the unevenness induced by the laser irradiation. However, all visible cells were vital (green) (Figure 4). Additional images of two specimens of the laser-welded membrane are shown in Supplementary Figure S1 to demonstrate that the cells are attached and alive in different focal planes of the membrane.

## 3. Discussion

In this interdisciplinary study, a team of engineers, surgeons, and biologists worked together to assess the feasibility of a novel approach for sutureless oral wound treatment. We could show that all utilized materials and processes are cytocompatible and the achieved bonding-strengths of the wound cover are sufficient for LTS on intraoral tissues.

Today, there is a high incidence of intraoral wound defects which can reach up to 42% of the population [36]. Until now, the gold standard treatments for large mucosal defects are distant or local flaps such as split and full-thickness skin grafts, oral mucosa free grafts, or oral connective tissue grafts [1,2,3]. However, these treatment options are associated with different side effects and varying success rates [3]. Especially worth mentioning is the limited supply of donor tissue and the associated donor site morbidities such as infections, bleeding, and wound healing disturbances. Thus, intraoral surgeries lead to long hospital stays and inconveniences for the patients and high health care costs.

The main disadvantages of classical suturing in the oral cavity are its technical difficulty and the time-consuming procedures resulting from difficult to reach anatomical sites and fragile tissue conditions. Moreover, the integrity of the oral mucosa is often compromised in elderly patients and patients with preexisting morbidities such as diabetes or vitamin deficiencies. Especially in those with periodontitis, which occurs in up to 50% of the global population [37]. Therefore, there is a great demand for procedures that circumvent these disadvantages.

One such procedure, laser-assisted repair, offers simplification of the intraoral surgery and reduction of the operation time, immediate watertight closure, and an optimum healing process. Recent advancements of laser-assisted tissue repair include the addition of dressing items as biopolymer or protein membranes or scaffolds to strengthen the seal both during and after the laser application [38]. 

However, until today there is no LTS system commercially available that allows for heat-regulated fixation of intraoral wound dressings. Following laser irradiation, the dressing materials are thermally forced to act as adhesives or glues that can form an interdigitated matrix with main tissue components such as collagen fibers or mucopolysaccharides. The disadvantage of overheating and the resulting tissue damage during laser-assisted tissue repair still discourages its application in oral surgery.

Several studies evidenced that the inhomogeneous and scarce control over distribution of the temperature can cause detrimental effects to tissue components like carbonization and other irreversible processes [39,40]. Consequently, the next step for a successful control of the whole process is an accurate monitoring procedure of the tissue exposure to laser irradiation in order to obtain enough mechanical strength between welded tissues and wound dressings while avoiding overheating damages. In other words, a real-time dosimetry of laser irradiation and of the corresponding temperature rise is crucial to minimize the risk of thermal damage to the tissue and to generate strong welds.

Wound patches based on collagen are the first option as substitute materials for this indication [4]. In this context, it has been shown that collagen-based biomaterials can support wound healing and epithelial regeneration in addition to their ability to minimize scarring and their barrier functionality [1]. Additionally, to the application of such wound patches it is well known that large mucosal defects require for specialized and complex suturing techniques that may lead to treatment errors and that are very time-consuming [5,7,8,9]. Our system includes a laser device and a transparent collagen membrane to be used as a wound patch. In this context, albumin as a serum protein was used as a bio-adhesive as it has already been described to be sufficient for such applications [24,25]. By integrating sensors which control the photothermal process in real-time, the newly developed laser system is expected to provide a reliable and easy-to-use device for quick and precise sutureless intraoral wound closure.

Further advantages of our laser-assisted sutureless wound-closure approach include the circumvention of bacterial translocation to the bloodstream, which can occur during intraoral conventional suturing, particularly in patients with heavy periodontitis. Recent publications in this field [41,42] highlight the connection between periodontitis and cardiovascular diseases, e.g., caused by progenitor cells. These associated problems might be avoidable by the use of intraoral LTS.

The aim our translational study was to test the feasibility of the approach and the cytocompatibility of the components of this completely new treatment option. The results of the initial ex vivo feasibility study revealed that the laser-assisted sutureless wound-closure on pork cheek led to a perpendicular tensile strength of 1.2 N. We were able to replicate this result for porcine palate with a slightly higher perpendicular tensile strength of 1.5 N. Notably, this was achieved with a held laser handpiece that was used manually by a surgeon where movement is unavoidable.

Compared to studies using similar approaches, these values are in a comparable range. For repairing skin defects via tissue laser soldering in pigs, a repair strength of ~1 N was shown [43], or lower, as shown for porcine skin with >30 g (corresponding to >0.29 N) [23].

The cytocompatibility assessments showed that both the membrane and the bioadhesive with and without laser irradiation were fully cytocompatible. The results showed that both the viability and proliferation of fibroblasts cultured in media incubated for 3 days with the membrane/bio-adhesive with and without laser irradiation were comparable with those of cells grown in media without contact to the materials. Thus, the results of this indirect test revealed a lack of release of toxic components from the collagen membrane and the bio-adhesive, regardless of laser-irradiation. In concordance, no significant cytotoxicity was measured for any of the specimens. When seeded directly onto the laser-irradiated collagen membrane with the bio-adhesive, the cells showed no signs of toxic effects. The observation that the cells appear rounder on the irradiated adhesive spots is due to the three-dimensional structure of the specimens.

Based on these in vitro results, it can be concluded that the newly developed collagen membranes as well as the complete fixation system are not eliciting undesired cell reactions. Further animal and ultimately clinical studies are necessary to gain a better understanding of this novel wound healing approach and to assess whether it is suitable for clinical routine use.

## 4. Materials and Methods

### 4.1. Transparent Pericardium-Derived Collagen Membrane

**Figure 5:**
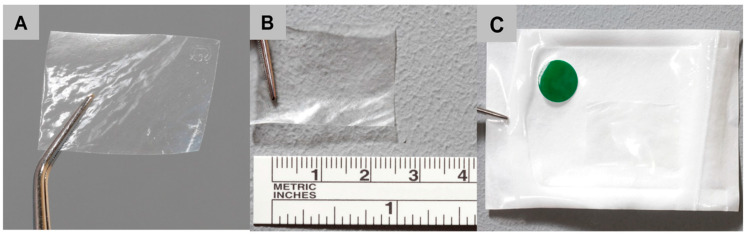
Image of the newly developed transparent collagen membrane (**A**–**C**).

The bovine pericardium collagen membrane was developed on basis of a commercially available barrier membrane (Jason membrane, botiss biomaterials GmbH, Zossen, Germany) via a new chemical treatment step using formic acid that was included in the decellularization process. The preparation of the utilized membrane is described in more detail by Gueldenpfennig et al. [31]. During the development process, both dermis- and pericardium-derived membranes were tested for their suitability to become transparent. In the end, only the pericardium showed the desired degree of transparency needed for laser fixation (Figure 5). Moreover, the resistance against collagenases was tested for pericardium derived membranes with the result that the pericardium-derived materials showed the desired degree of resistance [31]. The micro-structure of both membranes was imaged by scanning electron microscopy (SEM) using a Crossbeam 340 (Zeiss, Oberkochen, Germany). Prior to imaging, membranes were cut with a scalpel to reveal their cross-sectional plane, and sputtered with gold to enhance conductivity. Imaging was performed in SE mode at an acceleration voltage of 2 kV. 

### 4.2. Bio-Adhesive

Dried bovine serum albumin (BSA, Sigma Aldrich, Darmstadt, Germany) was dissolved in sterile phosphate buffered saline (PBS) without CaCl_2_ and MgCl_2_ prepared from a 10× concentrated stock (Gibco, Darmstadt, Germany) at a concentration of 42% (*w*/*w*). This was achieved by adding the BSA in 6–7 portions to the PBS under constant agitation by a magnetic stirrer (80 rpm, 40 mm agitator) during 6 h at room temperature, followed by an overnight incubation step at room temperature for clarification. The resulting bio-adhesive solution was stored at 4 °C until it was used.

### 4.3. Laser Construction

**Figure 6:**
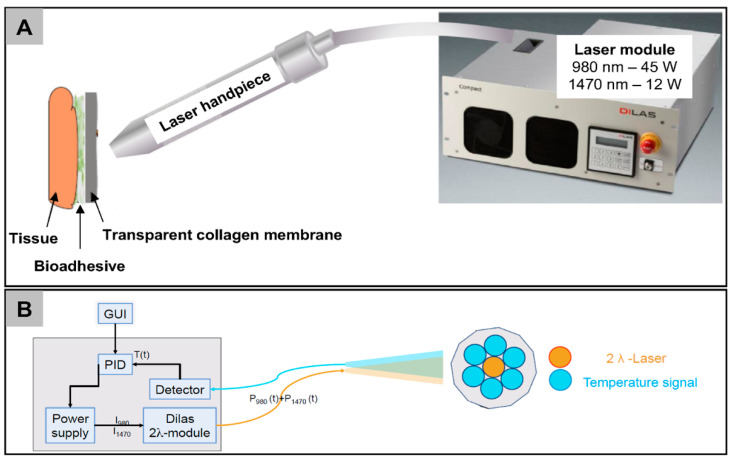
Utilized system for laser-assisted sutureless wound closure. (**A**) Illustration of the principle and the laser device. The laser beam is directed onto the bio-adhesive spots which then weld and bind the membrane and the underlying tissue together. (**B**) The laser-fiber composed of an aiming beam, a thermo-sensor, and a 2λ laser.

The experimental laser setup consists of a fiber-coupled double-bar laser diode module (DILAS type M1F4S22) equipped with 980 nm and 1470 nm laser diode bars, and a controller with two power supplies, and a built in temperature sensor that was included in the handpiece (Figure 6). The 980 nm and 1470 nm laser bars provide maximal output powers of 45 W and 12 W, respectively. The radiation of both bars is coupled into the same optical fiber with a core diameter of 400 µm, and a spacer was used to adjust the spot diameter on the tissue to 4–6 mm (Figure 6). The surface temperature is monitored by guiding the infrared radiation via a bundle of optical fibers to an infrared sensor (Optris CT3 ML, Optris GmbH, Berlin, Germany) with a response time of ~2 ms. The temperature signal is fed back to the laser controller which compares the measured temperature with a preset setpoint value for the target temperature in order to regulate the laser power accordingly. Upon initiation of the gluing process by pressing the laser footswitch, the laser emits initially a high preset laser power, which is then regulated down via the temperature signal. The process is stopped via a timer which had been set to 6 s in these experiments. The device is equipped with the usual safety measures of a medical laser device. The parameters that were used for conducting LTS in this study for the ex vivo feasibility tests were established by testing various parameter combinations and measuring the coagalation depth as well as bond strength in chicken breast and porcine palate ex vivo in an iterative process.

### 4.4. Ex Vivo Feasibility Tests

Fresh porcine cheeks and a fresh porcine palate were obtained from a local butcher in accordance with the local animal experiments committee. 

**Figure 7:**
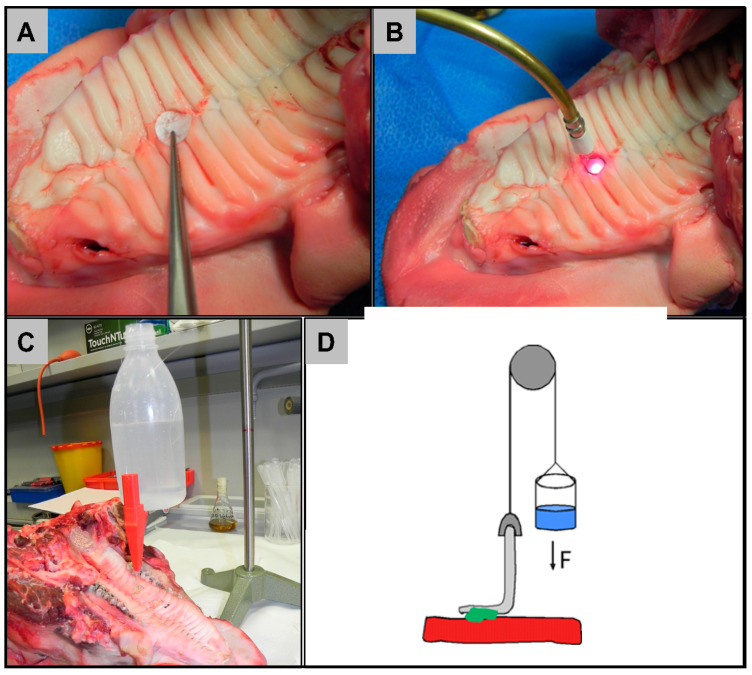
Procedure of the ex vivo laser welding tests. (**A**) Placement of the collagen membrane on top of the serum spots covering the wound. (**B**) Laser-welding using the laser hand-piece. (**C**) Measurement of the tensile strength. (**D**) Illustration of the principle of tensile strength measurements.

In a first step, the general feasibility of LTS of oral tissue was tested in a stationary experiment on porcine cheeks. In order to exclude the possibility that stationary application of the laser without any movement in the laser-beam reaches higher tensile strength values than clinically achievable in the oral cavity, the LTS process was validated using a laser that was equipped with a handpiece that was used freehandedly by the surgeon on porcine cheeks and subsequently on a porcine palate. For the tests on porcine palate, a round lesion of 2 mm depth and a diameter of 1 cm was prepared on the surface of the palate (Figure 7A,B). Around the lesion, the bioadhesive solution (described above) was spotted on six locations using a plastic pipette. The transparent collagen membrane was then placed on the serum spots to cover the lesion (Figure 7A). Laser radiation of 980 nm, 1470 nm, or both was applied on the spots of the bovine serum and activated for 6 s (Figure 7B). For measuring the bond-strength, we followed the protocol that was established by Steinsträsser et al. [23]. Immediately after the laser-welding fixation, the bond-strength between the collagen membrane and the tissue was measured in perpendicular tearing force (Figure 7C,D). Briefly, the whole porcine palate was held firmly on a bench and the membrane was clamped at a corner. One end of a thread was fixed to the clamp and the other end was threaded through a deflector and then fixed to a bottle. Water was filled into the bottle at a rate of approximately 3 mL per second. The minimum volume of water needed to tear off the membrane from the tissue is the tensile strength.

### 4.5. Cell Culture

L-929 mouse fibroblasts (LGC Standards, Wesel, Germany) were cultured in MEM (Minimum Essential Medium) supplemented with 10% fetal bovine serum, penicillin/streptomycin (100 U/mL each) (all from Life Technologies, Carlsbad, CA, USA) and l-glutamine at 37 °C, 5% CO_2_, and 95% humidity.

### 4.6. In Vitro Cytocompatibility Assessment

Assays were carried out as previously described [34]. For the extract assays, pieces with the dimensions of 1.5 × 1 cm collagen membranes with bio-adhesive, with and without laser-irradiation were incubated in 1 mL medium for 72 h to extract potential toxic components. Subsequently, the medium was used to culture cells for 24 h. On the next day, viability, proliferation and cytotoxicity were measured using respective XTT, BrdU, and LDH assay kits as previously described [34]. All three in vitro assays were carried out in four replicates for each test material. Means and standard deviations were calculated and normalized to the respective value of the non-toxic control. 

For the live-dead staining assay, a 1 × 1 cm piece of the collagen membrane with bio-adhesive, with and without laser irradiation were placed in one well of 24 well plates to which 1.2 × 10^5^ cells and 1 mL medium were added. RM-A and Wako plastic sheets were used as a toxic and a nontoxic control, respectively. Cells were incubated for 24 h and subsequently subjected to live–dead staining with propidium iodide and fluorescein diacetate. Green living cells and red dead cells were observed and evaluated under a fluorescence microscope.

### 4.7. Reference Materials and Controls for the In Vitro Cytocompatibility Assessment

We used controls and reference materials as recommended in ISO 10993-5 [35]. As a toxic control, RM-A (Hadano Research Institute, Food and Drug Safety Center, Hadano, Japan) was used for all assays. For the extract assays, medium incubated without test material was used as am established nontoxic reference material (negative control). For the live–dead staining assay, Wako plastic sheets (Wako Pure Chemical Industries, Ltd., Osaka, Japan, cat. no. 160-08893) were used as a nontoxic control.

### 4.8. Statistical Analysis

Statistical analysis was performed using the software Graphpad Prism 5 (GraphPad Software, Inc., La Jolla, CA, USA). All groups were tested for normal distribution with the Kolmogorov–Smirnov test. The difference between fixation using a stationary laser and fixation using a laser equipped with a handpiece was analyzed using an unpaired two-tailed *t*-test. *p*-Values < 0.05 were considered statistically significant. 

## 5. Conclusions

In conclusion, the newly developed laser device in combination with a transparent collagen wound patch could be a novel feasible option for faultless and easy wound closure of large mucosal defects. The combination of the transparent collagen membrane and the laser-assisted wound closure shows sufficient tensile strengths in an ex vivo pig palate model and suitable cytocompatibility. Nonetheless, this work should primarily be considered as a feasibility study and a first step on the way towards a laser-based sutureless clinical application in tissue repair and oral surgery. Extensive further testing—e.g., on an animal models—is needed to obtain more understanding of its suitability in vivo.

## Supplementary Materials

The following are available online at https://www.mdpi.com/1422-0067/21/19/7104/s1, Figure S1: L929 cells grown on the laser-welded membrane + bioadhesive.
