# Supplementary material for: In Vitro Feasibility Analysis of a New Sutureless Wound-Closure System Based on a Temperature-Regulated Laser and a Transparent Collagen Membrane for Laser Tissue Soldering (LTS)
Source: Int J Mol Sci. 2020 Sep 26;21(19):7104. doi: 10.3390/ijms21197104 (PMC7582393; doi:10.3390/ijms21197104)
Supplement: Supplementary file 1 [file ijms-21-07104-s001.pdf]

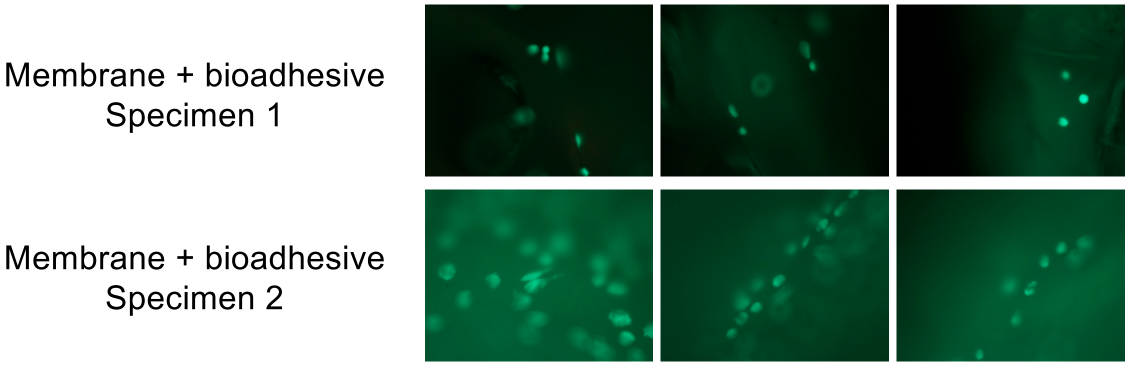

Supplemental Figure S1. L929 cells grown on the laser-welded membrane + bioadhesive. Vital and dead cells show green and red fluorescence, respectively. Three different areas of each of two specimens of the laser-welded membrane are shown to demonstrate that the cells are attached and alive in different focal planes of the membrane.
